# The Social Explanatory Styles Questionnaire: Assessing Moderators of Basic Social-Cognitive Phenomena Including Spontaneous Trait Inference, the Fundamental Attribution Error, and Moral Blame

**DOI:** 10.1371/journal.pone.0100886

**Published:** 2014-07-09

**Authors:** Michael J. Gill, Michael R. Andreychik

**Affiliations:** 1 Department of Psychology, Lehigh University, Bethlehem, Pennsylvania, United States of America; 2 Department of Psychology, Fairfield University, Fairfield, Connecticut, United States of America; University of New South Wales, Australia

## Abstract

*Why is he poor? Why is she failing academically? Why is he so generous? Why is she so conscientious*? Answers to such everyday questions—*social explanations*—have powerful effects on relationships at the interpersonal and societal levels. How do people select an explanation in particular cases? We suggest that, often, explanations are selected based on the individual's pre-existing general theories of social causality. More specifically, we suggest that over time individuals develop general beliefs regarding the causes of social events. We refer to these beliefs as *social explanatory styles*. Our goal in the present article is to offer and validate a measure of individual differences in social explanatory styles. Accordingly, we offer the Social Explanatory Styles Questionnaire (SESQ), which measures three independent dimensions of social explanatory style: Dispositionism, historicism, and controllability. Studies 1–3 examine basic psychometric properties of the SESQ and provide positive evidence regarding internal consistency, factor structure, and both convergent and divergent validity. Studies 4–6 examine predictive validity for each subscale: Does each explanatory dimension moderate an important phenomenon of social cognition? Results suggest that they do. In Study 4, we show that SESQ dispositionism moderates the tendency to make spontaneous trait inferences. In Study 5, we show that SESQ historicism moderates the tendency to commit the Fundamental Attribution Error. Finally, in Study 6 we show that SESQ controllability predicts polarization of moral blame judgments: Heightened blaming toward controllable stigmas (assimilation), and attenuated blaming toward uncontrollable stigmas (contrast). Decades of research suggest that explanatory style regarding the self is a powerful predictor of self-functioning. We think it is likely that social explanatory styles—perhaps comprising interactive combinations of the basic dimensions tapped by the SESQ—will be similarly potent predictors of social functioning. We hope the SESQ will be a useful tool for exploring that possibility.

## Introduction

Constructing explanations is vital for the human comprehension of reality. Furthermore, as social beings our explanatory abilities are often aimed at understanding other people. *Social explanations* are the product of this social sense-making activity. *Why is he helpful to strangers? Why is she so successful*? Our answers to such everyday questions have powerful effects on interpersonal relationships [1], [2], moral emotions [3]–[8], reactions to success and failure [9], [10], intergroup relations [11]–[14], and marital quality [15]–[17]. It seems that all major domains of social life are shaped by patterns of social explanation.

Here, we are interested in the possibility that individuals have enduring, characteristic styles of social explanation, which we call *social explanatory styles* [18], [19]. Given the centrality of explanation to social life, social explanatory styles should have broad implications for the social life of the individual, pervasively shaping her cognitive, emotional, and behavioral responses to others. Indeed, a large body of work suggests that characteristic styles of explaining one's *own* acts and outcomes have profound effects on individual adaptation [20]–[22]. We expect that social explanatory styles have similarly potent implications for social functioning.

Below, we offer a measure of individual differences in social explanatory styles: The *Social Explanatory Styles Questionnaire* (*SESQ*). The measure taps three basic explanatory dimensions (described below). To validate the measure, we will provide evidence that each of these basic dimensions moderates an important phenomenon of social cognition: Spontaneous trait inferences [23], the fundamental attribution error [24], and blaming of those with stigmatizing conditions [7]. Although we focus here on the preliminary task of separately validating each dimension of the SESQ, we encourage researchers to consider interactions among these basic dimensions as they try to illuminate particular social judgment phenomena. Prior to presenting evidence regarding the reliability and validity of the SESQ, we will situate the construct of social explanatory styles it in the literature in two ways. First, we will show how our conception of social explanatory styles grows out of classic work regarding the conceptual structure of social explanations. Second, we will elaborate on how our approach is rooted in a particular view of the social explanatory process.

## The Conceptual Structure of Social Explanations

Heider [1] pioneered the image of everyday social life as suffused by causal analysis. He also offered seminal ideas about the conceptual structure of lay thinking about social causality. In particular, Heider noted that lay perceivers view others as both “*affected* by their personal and impersonal environment” and as able to “*cause* changes in the environment” (p. 17; italics in original). This distinction between being “affected by” versus “causing” is seen in the longstanding theoretical distinction between *external* (or *situational*) *causes*, which involve personal and impersonal forces that surround an actor and shape her acts or outcomes, and *internal* (or *dispositional*) *causes*, which involve personal abilities, motives, or traits that reside within the actor. This distinction takes center stage in the majority of empirical work on social explanation [25]; but see [26] for an argument that this distinction should not take center stage. Although early work assumed that internal and external causes are mutually exclusive causal choices, research has undermined this assumption [27]; for in-depth discussion see also [28]. Perhaps the most powerful argument against an internal/external dichotomy is that external forces can be seen as creating internal dispositions [e.g., *He lacks confidence* (internal) *because his parents were very critical* (external).]. Thus, our conception of social explanatory styles treats internal and external causes as independent dimensions.

Weiner [7]–[10] played a major role in pushing psychologists to think beyond internal and external causes. Most famously, Weiner highlights the causal property of *controllability*. For example, according to Weiner and Kukla [9], evaluations of others' achievements are shaped by the perceived causal role of internal-controllable (*effort*) versus internal-uncontrollable (*intelligence*) causes. Controllable failures evoke blame and anger, whereas uncontrollable failures evoke compassion. Broadly speaking, controllability perceptions are high when the perceiver thinks that volitional activity by the actor could have made things turn out otherwise and low when the actor is seen as having little volitional control over how things turned out. Weiner [10] also highlighted the notion of causal *stability*: Some causes are constant (traits), whereas others fluctuate (moods). We included the stability dimension in our research for quite some time, but found no evidence that it mattered for the phenomena we studied. Thus, we will not discuss it further.

## The Process of Social Explanation

### From Naïve Scientist to Cognitive Miser

In addition to focusing on conceptual structure, classic work also attempted to describe how perceivers select an explanation [29], [30]. Classic models focused on how perceivers choose between internal and external causes, and portray perceivers as engaged in logical analysis like a *naïve scientist*. Furthermore, classic models portray the explanatory process as largely “bottom up,” assuming that explanations are selected based on case-specific observations regarding the particular actor and act being explained [25]. For example, Jones and Davis [29] proposed that perceivers select an internal explanation if they answer affirmatively to the question: *Did the actor intentionally bring about effects that most people would try to avoid*? Kelley [30] proposed that internal explanations are selected when the perceiver answers affirmatively to three questions: *Has the actor previously acted like this in similar contexts? Does the actor act like this in many contexts? Is the act different from what others would do*?

Although these models contain some truth [31], research has also uncovered important shortcomings. Most famously, Jones and Harris [24] found that perceivers select internal causes even when classic models suggest it is logically inappropriate to do so (i.e., when there is an obvious external cause present). This pervasive bias has been dubbed the *Fundamental Attribution Error* [32] or the *Correspondence Bias* [27]. Gilbert, Pelham, and Krull [33] illuminated a major cause of the FAE. They presented evidence that perceivers automatically generate internal explanations for acts, and only consider external causes if they are both motivated and able to do so [34], [35]. This work frames the perceiver as an unscientific *cognitive miser* who prefers to exert as little cognitive effort as possible. The miser “prefers” internal explanations because they are easy to generate [23], [36].

### The Lay Theories Perspective: Perceiver as *Meaning-Maker*

An alternative perspective—reflected in our concept of social explanatory styles along with other work to be discussed below—is that explanation selection is guided by the perceiver's preexisting theories. This *lay theories perspective* assumes that perceivers vary in their general beliefs and expectations regarding social causality. The starting point for the lay theories perspective is the overwhelming complexity of the social world, the vast array of social events one must explain daily and the vast array of causes that might potentially have given rise to each of those events. In the face of such complexity, pre-existing theories—general beliefs and expectations about “how things typically work”—are indispensable for making sense of it all. Although theories do render the world easier to manage cognitively, the lay theories perspective asserts that needs for *understanding* and *meaning*—not miserliness—are the primary motives underlying theory development [1], [37], [38], [39], [40]. After building a theory, perceivers are not open-minded but rather tend to automatically see the world in terms of their theories. Our major prediction, rooted in this lay theories perspective, is that several well-documented social-cognitive phenomena will be moderated by individual differences in theories of social causality [39]–[42].

The existing literature contains two major lines of work reflecting a lay theories perspective. The first examines cultural differences. For example, Miller [43] found that Americans show a preference for internal explanations, whereas Indians show a preference for external explanations. Similar results have been reported by Morris and Peng [44]. Various challenges have been offered to the simplistic idea that Westerners prefer internal whereas Easterners prefer external causes. For example, some work has suggested that cultural differences emerge only when external causal information is highly salient, in which case Easterners use it and Westerners continue to ignore it [45], [46]. More subtly, Menon, Morris, Chui, and Hong [47] suggest that Westerners and Easterners both emphasize internal causes, but Easterners are more likely to see dispositions residing in groups (*the organization* is corrupt) whereas Westerners are more likely to see dispositions as residing in individuals (*he* or *she* is corrupt). In spite of these nuances, the literature is clear that there are cultural differences in general theories of social causality, which guide explanation selection in specific cases.

Beyond this work on culture, the best-known approach to lay theories comes from Dweck and her colleagues' work on implicit person theories [39], [40], [48], [49]; see also [50] for a distinct, more recent approach. They take an individual differences (rather than cultural) approach and suggest that lay perceivers can be classified either as *entity theorists*, who believe that the characteristics of human beings are highly stable (“Everyone is a certain kind of person and there is not much that can be done to change that”), or *incremental theorists*, who believe that human characteristics are malleable and unstable (“All people can change even their most basic qualities”). One of the clearest demonstrations of the effects of implicit person theories comes from Chiu, Hong, and Dweck [51]. They found that entity theorists were more likely than incremental theorists to believe that an actor's behavior in one context would predict his behavior in a different context and that even a single instance of behavior is indicative of an enduring behavioral tendency. Other work [Hong, 1994, unpublished] has suggested, in contrast, that incremental theorists favor explanations that point to dynamic processes in the actor's mind (e.g., momentary needs, goals, or strategies), which create expectations of less behavioral stability.

More recently, Church and his colleagues [50] proposed an alternative conceptualization of lay theories. First, they offer the concept of *implicit trait beliefs*, which are a person's beliefs about the stability of traits over time, the consistency of trait-relevant behavior across situations, the ease of predicting behavior from traits, the ease of inferring traits from scant behavioral observations, and the adequacy of traits for understanding a person. They contrast implicit trait beliefs with *implicit contextual beliefs*, which are a person's beliefs about the instability of traits over time, the inconsistency of trait-relevant behavior across situations, the inability to predict specific behaviors from traits, the difficulty of inferring traits from scant behavioral observations, and the importance of context (e.g., roles, statuses) for predicting behavior. Church et al. developed the *Personal Beliefs Inventory* (*PBI*) to measure these two dimensions of lay theories. They found that the PBI comprised two underlying latent factors, which corresponded to implicit trait and implicit contextual theories. This, of course, provides additional evidence regarding the orthogonality of internal and external casual explanations noted above. Furthermore, Church et al. presented evidence that implicit trait beliefs were only weakly related to Dweck et al.'s implicit person theories, and implicit contextual beliefs were not related to them at all.

Our concept of social explanatory styles has much in common with this prior work. How will we contribute beyond it? First, our focus on individual differences distinguishes our approach from work on culture. Second, although Dweck et al. and Church et al. focus on individual differences, we conceive of social explanatory styles in terms of three independent dimensions, only one of which seems likely *a priori* to be empirically related to their constructs (and which we will show below is only moderately related to Church's implicit trait beliefs and is unrelated to Dweck et al.'s theories). To elaborate, unlike the SESQ, Dweck et al. do not directly measure belief in the efficacy of external causes. This is important given the arguments above that such belief is orthogonal to belief in the efficacy of internal causes (which Dweck et al. do measure). Relatedly, unlike the SESQ, Church et al. do not measure belief in the efficacy of actor volition/control. Finally, whereas Church et al. do measure belief in the efficacy of external causes, they conceive of external causes as *contextual* influences that cause an actor to behave differently on different occasions. In contrast, as will be elaborated below, we conceive of external causes as *historical* and *formative* influences that have caused an actor to *become a particular kind of person*. Our conception grows out of our research on moral psychology, which suggests that moral emotions such as blame and compassion are linked to beliefs about the role of historical, formative influences in shaping potentially blameworthy agents [4], [11]. To maintain clarity, we will use more descriptive labels such as *historicism* and *historical causes* when discussing the types of external causes assessed by the Social Explanatory Styles Questionnaire. The upshot here is that, in relation to existing lay theories measures, the SESQ provides predominantly novel information. We will provide evidence below regarding the relation between the SESQ, Dweck et al.'s implicit person theories measure, and Church et al.'s PBI.

## Social Explanatory Styles

What are social explanatory styles and where do they come from? We propose that, over time, cultural influences, individual learning experiences, and individual cognitive and motivational influences combine to create individual differences in general beliefs about the causes of action. We propose that important individual differences exist in general beliefs about the causal importance of internal dispositions, historical, formative influences in an agent's life, and agent volition/control. Thus, we conceptualize social explanatory styles in terms of three independent dimensions: *Dispositionism*, *historicism*, and *controllability*. Although we suspect that these different dimensions likely interact to predict certain social judgments, in the present paper we focus on the preliminary task of separately validating our measure of each independent dimension.

Individual differences in social explanatory styles are likely shaped by a wide array of factors. At the broadest level, cultures vary in terms of beliefs about social causality (see above). There is also evidence for ethnic group differences in causal perceptions within the United States [52]. At a more specific level, educational experiences likely shape social explanatory styles. For example, higher education can reduce internal explanations [53] and increase reliance on more sophisticated “interactionist” explanations [52]. Higher education in the social sciences strengthens belief in historicism, at least for understanding group-level outcomes [54], [55]. Finally, at an intrapsychic level, those who are contemplative are relatively likely to adopt a belief in historicism [18]. Surely, much future work is needed to understand the full array of factors that create variability in social explanatory styles.

Understanding the causes and consequences of diversity in social explanatory styles will be greatly facilitated by the availability of a reliable and valid measure. Thus, below we present six studies that examine the convergent and divergent validity of the Social Explanatory Styles Questionnaire. As noted above, given the central role of explanation in social life, individual differences in social explanatory styles—likely involving interactive relations among the independent dimensions tapped by the SESQ—should have broad implications for the individual's social life. That assumption provides the motivation for the present work.

### Measuring Social Explanatory Styles

Beyond the work of Dweck et al. and Church et al., other scholars have developed instruments that are similar in some ways to what we are attempting. For example, Fincham and Bradbury [16] developed the Relationship Attribution Measure (RAM). Research using the RAM shows that explanatory styles in the marital context plays a role in shaping marital quality. But, the RAM does not tap broader explanatory tendencies that generalize beyond one's marriage. Relatedly, Peterson, Semmel, von Baeyer, Abramson, Metalsky, and Seligman [21] developed the Attributional Style Questionnaire (ASQ). The ASQ taps *explanatory style*, or one's characteristic style of explaining one's own acts and outcomes [56]. Work with the ASQ has suggested the existence of a *depressive attributional style*: Those who attribute negative personal events to their own stable, global dispositions (i.e., an interactive combination of belief in internality, stability, and globality) are at increased risk of depression. The notion of explanatory styles is quite similar to our notion of social explanatory styles, and its role in predicting depression speaks to the importance of general explanatory theories. The major difference, of course, is that explanatory styles concern the self, whereas social explanatory styles concern others.

To measure our construct, over a period of years we have developed the SESQ, which can be seen in Appendix S1. Respondents are presented with descriptions of the action patterns of eight different individuals. Each of the eight scenarios was designed to describe the general character of a target. That is, the SESQ taps explanations regarding the enduring character of a person rather than explanations for single, isolated acts. Half the targets show positive character and half show negative character. For each target individual, respondents are asked: *Why has [this target] become [this way]*? They provide ratings of the extent to which the cause is the target's traits (*dispositionism*), the target's external circumstances/life experiences (*historicism*), and factors over which the actor can exert control (*controllability*). Scoring of the SESQ involves calculation of mean dispositionism, historicism, and controllability scores.

## The Present Studies

### Ethics Statement

All studies were approved by Lehigh University's Institutional Review Board (IRB). For each study, participants provided written consent. The consent forms were approved by Lehigh University's IRB.

### Overview of Studies

Below, we present six studies. Studies 1, 2 and 3 will examine basic psychometric properties of the SESQ. We will present evidence regarding the internal consistency of responses to each dimension of the SESQ, factor structure of the SESQ, relation to personality traits and political ideologies, and relation to the lay theories measures of Dweck et al. and Church et al.

Studies 4, 5, and 6 address predictive validity. The approach in each study is to validate a particular subscale of the SESQ. As noted above, however, we encourage users to examine interactions among SESQ dimensions for predicting social judgments and behaviors. Such interactions will be more meaningful to the extent that the contributing dimensions from the SESQ have been separately validated. Thus, such preliminary validation is the task of the present studies. Study 4 will test whether SESQ dispositionism moderates the tendency to make spontaneous trait inferences from behaviors [23]. Study 5 will test whether SESQ historicism moderates the tendency to commit the FAE in the classic Jones and Harris [24] attitude attribution paradigm. Study 6 will examine whether SESQ controllability moderates blaming of those from stigmatized categories [7]. Taken together, these studies will show that social explanatory styles play an important role in moderating several basic phenomena of social cognition and thus that each separate subscale of the SESQ has an important degree of predictive validity.

## Study 1: Internal Consistency and Factor Structure of the SESQ

### Method

#### Participants

Three-hundred and ten participants (184 males, 126 females) participated on-line via the Mechanical Turk interface created by Amazon. They ranged in age from 18 to 68 (*M* = 29.5). Many participants had completed a 4-year college degree (35%) and some had also completed an advanced professional or graduate degree (8%). Other participants had completed a 2-year degree (11%), less than two years of college (30%), or a high school degree (15%). A few participants (1%) had not completed high school. The sample was predominately White (87%), but also included individuals who identified as East Asian, African-American, and Hispanic.

#### Procedure

After clicking the “agree” box on an on-line consent form, participants completed the SESQ along with some unrelated measures.

### Results and Discussion

We began by examining internal consistency for each scale dimension. Cronbach's alpha suggested strong internal consistency for dispositionism (α = .89), historicism (α = .85), and controllability (α = .88). These support the idea that people have coherent social explanatory styles. Next, we submitted mean ratings on each dimension to a repeated-measures ANOVA. This revealed a strong effect of causal dimension, *F*(2, 308) = 113.11, *p*<.001. The pattern was such that controllability ratings were the highest (*M* = 4.37, *SD*  = .68), dispositionism ratings were next (*M* = 3.98, *SD*  = .64), and historicism ratings were lowest (*M* = 3.51, *SD*  = .66). Controllability ratings were significantly higher than both dispositionism and historicism ratings (*F*s>46.96, *p*s <.001, *d*s>.78), and dispositionism ratings were significantly higher than historicism ratings (*F* = 67.47, *p*<.001, *d* = .94). This pattern fits the notion that those in Western cultures tend to view actions as highly controllable [57] and to emphasize internal over external causes [43], [44]. Of course, these mean differences should be interpreted cautiously given that we did not obtain data from a comparison culture to see if the pattern of means would change. Finally, we examined correlations among SESQ dimensions. Supporting our decision to measure them separately, dispositionism and historicism were unrelated, *r*(308)  = .08, *ns*. In contrast, dispositionism and controllability showed a modest positive relation, *r*(308)  = .19, p<.001, and historicism and controllability showed a weak negative relation, *r*(308)  = −.11, p<.05.

Finally, we computed a principal components factor analysis with varimax rotation. Looking at the scree plot, it was clear that a three factor solution described the data quite well. The eigenvalues of these three factors were 5.77, 4.45, and 3.18, and together they accounted for 56% of the variance. There were two additional factors with eigenvalues between 1.0 and 1.2 but these factors had just one or two items with factor loadings over |.50|. Thus, the structure of the SESQ is dominated by three factors. These three factors are displayed in Table 1. Clearly, each causal dimension tapped by the SESQ—dispositionism, historicism, controllability—represents a separate underlying latent factor. Thus, the SESQ reliably measures three independent facets of people's social explanatory styles.

**Table 1:** Factor Analysis of the SESQ (Study 1).

Item	Factor 1	Factor 2	Factor 3
Dispositionism: Susan's Love	.78		
Dispositionism: Bill's Helpfulness	.78		
Dispositionism: Steven's Disagreeableness	.75		
Dispositionism: Robert's Crimes	.74		
Dispositionism: James's Community Service	.74		
Dispositionism: Sarah's Verbal Abuse of Children	.73		
Dispositionism: Janet's Volunteering	.72		
Dispositionism: Beth's Infidelities	.71		
Control: Janet's Volunteering	.33	.85	
Control: James's Community Service		.84	
Control: Bill's Helpfulness		.80	
Control: Susan's Love		.79	
Control: Beth's Infidelities		.56	
Control: Steven's Disagreeableness		.48	
Control: Robert's Crimes		.42	
Control: Sarah's Verbal Abuse of Children		.35	
Historicism: Bill's Helpfulness			.81
Historicism: James's Community Service			.81
Historicism: Janet's Volunteering			.79
Historicism: Susan's Love			.78
Historicism: Robert's Crimes			.57
Historicism: Sarah's Verbal Abuse of Children			.50
Historicism: Steven's Disagreeableness			.36
Historicism: Beth's Infidelities			.26
			

*Note*. Factor loadings (including cross-loadings) less than |.25| are deleted.

## Study 2: Relation of the SESQ to Cognitive Style, Personality Traits, and Political Ideology

Study 2 sought evidence regarding the relation of social explanatory styles to cognitive processing style, personality traits, and political ideology. We propose that social explanatory styles are specific social cognitive orientations and thus they should relate, at most, only modestly to broad personality traits. Beyond this, we expected that individuals prone to thorough cognitive processing would be more historicist and less dispositional in their social explanatory styles [18], [33]. Finally, we expected that social explanatory style would *not* be closely aligned with political ideology. With regard to this latter issue, there is evidence that liberals and conservative do show some differences in their thinking about social causality [58]–[61]. However, as we have argued elsewhere [18], these differences are specific to thinking about socio-economic inequality and do not reflect the type of broad explanatory theories we are trying to tap with the SESQ; see [60] for a similar perspective.

### Method

#### Participants

Two-hundred eighty undergraduate participants (145 female) participated for credit in their introductory psychology classes.

#### Procedure

After signing a written consent form, all participants completed the SESQ. In addition, a subset of sixty-seven completed the Need for Cognitive Closure scale (NFCC) [62], a separate subset of ninety-seven completed the Big Five Inventory (BFI) [63] and the Difficulties in Emotion Regulation Scale (DERS) [64], and another subset of one-hundred sixteen completed the Right-Wing Authoritarianism (RWA) [65] and Social Dominance Orientation scales (SDO) [66]. As per standard scoring methods, participants received a single score for NFCC [*M* = 3.49 (scale: 1 to 6), *SD* = .42, α = .83], DERS [*M* = 2.30 (scale: 1 to 5), *SD* = .64, α = .88], RWA [*M* = −1.09 (scale: −4 to 4), *SD* = 1.24, α = .92], and SDO [*M* = 3.00 (scale: 1 to 7), *SD* = 1.19, α = .94]. Also as per standard scoring methods, the BFI yielded five scores (scale: 1 to 5), with each score representing one of the Big Five Personality Factors: Extroversion (*M* = 3.25, *SD*  = .85, α = .88), Agreeableness (*M* = 3.67, *SD* = .53, α = .66), Conscientiousness (*M* = 3.52, *SD*  = .63, α = .79), Neuroticism (*M* = 2.90, *SD* = .72, α = .79), and Openness (*M* = 3.38, *SD* = .64, α = .77).

### Results and Discussion

Correlations were computed between the SESQ dimensions and the other constructs (see Table 2). We also computed multiple regression analyses. For all regression analyses presented in this paper, predictor variables were centered on their means prior to being multiplied to create interaction terms [67]. In the present case, we tested main effects and all 2- and 3-way interactions involving the SESQ dimensions. We found only one significant interaction effect out of 32 possible interactions, so we will treat that effect as spurious (it was not a highly sensible pattern).

**Table 2:** Correlations of SESQ dimensions with cognitive style, personality traits, and political ideology (Study 2).

	NFCC	DERS	Extroversion	Agreeableness	Conscientiousness	Neuroticism	Openness	RWA	SDO
	(*N* = 67)	(*N* = 97)	(*N* = 97)	(*N* = 97)	(*N* = 97)	(*N* = 97)	(*N* = 97)	(*N* = 116)	(*N* = 116)
Dispositionism	.40**	−.02	.13	.17	.04	−.05	.06	−.07	−.05
Historicism	−.27*	−.04	.14	.21*	.10	.05	.02	−.03	.06
Controllability	.23^†^	−.24*	−.09	.13	.29**	−.24*	−.25*	−.08	−.13

*Note*: ^†^ *p* = .06; *p<.05; **p<.01.

As can be seen in Table 2, the strongest correlations were between the SESQ dimensions and NFCC. Specifically, among those high in NFCC social explanatory styles tended to be more dispositional, less historicist, and marginally more likely to emphasize controllability. This is consistent with the notion that careful and thorough thinking increases one's focus on external forces such as agent history, whereas shallow thinking leaves one focused on internal factors such as dispositions and volition. Dispositionism was unrelated to all the other variables measured. In contrast, beyond its relation to NFCC, historicism was also modestly related to Agreeableness. This fits with the idea that historicism is associated with prosocial responding [18], [61]. Finally, controllability was associated with several variables beyond NFCC. In particular, controllability was negatively related to DERS and Neuroticism. It seems likely that these relations involve extrapolation from the self—*my emotions are strong and I have difficulty controlling them*—to a general belief about humanity—*people are not in control*. Also, those high in Openness were similarly likely to reject the idea that people are in control of their actions. This relation likely stems from an influence similar to that of NFCC: Contemplation tends to undermine belief in “free will.” Finally, in contrast, those high in Conscientiousness were more likely to score high on controllability. This, as with DERS and Neuroticism, seems likely to reflect extrapolation from the self—*I am consistently able to exercise self-control and enact appropriate behaviors*—to humanity—*people are in control of their actions*. None of the SESQ dimensions were related to political ideology.

## Study 3: Social Explanatory Styles, Implicit Person Theories, and Implicit Trait and Contextual Beliefs

In Study 3, we will examine the relation between the SESQ dimensions, Church et al.'s implicit trait and implicit contextual beliefs, and Dweck et al.'s implicit person theories.

### Method

#### Participants

One-hundred thirty-nine participants (76 female) participated for credit in their introductory psychology course. An additional one-hundred nineteen participants (87 males, 32 females) participated on-line via Amazon's Mechanical Turk interface. They ranged in age from 18 to 66 (*M* = 35). Many of the on-line participants had completed a 4-year college degree (51%) and some had also completed an advanced professional or graduate degree (10%). Other participants had completed a 2-year degree (10%), less than two years of college (17%), or a high school degree (12%). The sample was predominately White (81%), but also included individuals who identified as East Asian, African-American, and Hispanic.

#### Procedure

After providing consent, participants completed the SESQ. In addition, the introductory psychology sample completed Levy and Dweck's [unpublished] 8-item IPT measure (e.g., *Everyone is a certain kind of person, and there is not much they can really do to change that*). After appropriate reverse coding, items were averaged such that high scores reflected an entity theory (*M* = 4.0 of 7; *SD* = 1.3; α = .93). In scoring, we followed the procedure of Chiu et al. [51] by deleting those who scored around the midpoint (4) of the scale (who lack a clear theory). Specifically, we categorized those scoring over 4.5 as entity theorists and those scoring below 3.5 as incremental theorists. This resulted in the deletion of roughly 33% of cases, which is comparable to Chui et al. The on-line sample completed Church et al.'s [50] PBI measure, which taps implicit trait (*M* = 3.83 of 5; *SD* = .34; α = .88) and implicit contextual beliefs (*M* = 3.22; *SD* = .36; α = .86).

### Results and Discussion

We computed correlations between the three SESQ dimensions, IPT, and the two dimensions of the PBI. As can be seen in Table 3, there were no significant relations between the SESQ dimensions and Dweck et al.'s IPT measure. Indeed, there were only two significant correlations among the nine we computed. One of these—a positive relation between SESQ controllability and PBI implicit trait theories—was quite small. There was a somewhat larger relation between SESQ dispositionism and PBI implicit trait theories, although this correlation hardly suggests that the measures are redundant. Taken together, these correlations suggest that the SESQ is providing predominately novel information—not tapped by alternative measures—regarding lay theories of social causality. One task for the future will be to more precisely understand the differences among the constructs being assessed by these measures, a task that will become more achievable as more data are collected involving the newer measures (i.e., PBI, SESQ). Indeed, Church and colleagues [50] showed that the PBI was only weakly related to Dweck et al.'s IPT measure. In particular, then, further investigation is needed to more fully understand why the IPT measure, PBI implicit traits beliefs, and SESQ dispositionism are only weakly to moderately correlated, given their shared emphasis on traits/internal factors.

**Table 3:** Correlations of SESQ dimensions with Dweck et al.'s Implicit Person Theories and Church et al.'s Implicit Trait and Implicit Contextual Theories (Study 3).

	Dweck et al.: Implicit Person Theories	Church et al.: Implicit Trait Theories	Church et al. Implicit Contextual Theories
	(*N* = 93)	(*N* = 119)	(*N* = 119)
Dispositionism	.17	.40***	−.11
Historicism	−.01	.04	.07
Controllability	−.15	.20*	.09

*Note*: *p<.05; ***p<.001.

## Study 4: Dispositionism and the Tendency to Make Spontaneous Trait Inferences

Studies 1–3 provided strong evidence regarding the internal consistency, factor structure, and divergent validity of the SESQ. None of the evidence, however, demonstrates the predictive validity of each SESQ dimension. Thus, the purpose of the next three studies is to show that each dimension of the SESQ enables prediction of a relevant social psychological phenomenon. Study 4 will provide evidence that SESQ dispositionism moderates the tendency to make spontaneous trait inferences from behavior. Study 5 will provide evidence that SESQ historicism moderates the tendency to commit the Fundamental Attribution Error. Finally, Study 6 will show that SESQ controllability moderates blaming of those in stigmatized categories. As noted above, here we focus on the preliminary task of separately demonstrating the validity of each SESQ dimension. Future research will, we hope, consider whether interactions among particular social explanatory dimensions might be relevant for understanding a target phenomenon (e.g., as with depressive attributional style).

In Study 4, we utilize a probe recognition paradigm [68] to examine whether the tendency to make *spontaneous trait inferences* (*STI*s) is moderated by the dispositionism facet of social explanatory styles. Such a result takes us outside the realm of self-reports and would provide compelling evidence that dispositionism (as tapped by the SESQ) guides presumably automatic, uncontrollable social cognitive processes.

In the STI paradigm, participants read a series of one-sentence behavior descriptions which are followed by a probe word. Their task is to indicate as quickly and accurately as possible whether the probe word actually appeared in the behavior description. In reality, the probe word did not appear. On critical trials, however, the probe word is a trait (or situation) *implied* by the behavior description. On control trials the probe word is not implied by the behavior description. The logic behind this paradigm [69], [70, [71] is that if participants make a STI, then they should have difficulty correctly reporting that an implied probe word did not actually appear. Indeed, evidence for the occurrence of STIs comes when participants take longer to respond that a probe word (*smart*) did not appear on a critical trial (*John got an A on the test*) than on a control trial (*Ben jumped over the fence*). Such slow responding suggests that participants spontaneously inferred the probe word, an inference that interferes with correctly reporting that the word was not actually in the sentence. Ham and Vonk [70] expanded the STI paradigm to also measure s*pontaneous situation inferences* (*SSI*s). The logic is identical to that just described. For example, evidence for an SSI happens if, after reading *Eric lifted the boulder*, participants are slower to report that *light* was not in the sentence than after reading a control behavior description (*Steven swam the lake*). We use the stimuli from Ham and Vonk for our study below.

Our major prediction is that SESQ dispositionism scores will be positively related to the tendency to make STIs. Indeed, because lay theories presumably structure one's understanding of social events on a daily basis, those with strong dispositionist theories should be well-practiced at generating relevant trait concepts from behavioral observations. Thus, such inferences should be highly automatized and hence likely to be detectable in a STI paradigm. If our prediction is confirmed, it will suggest that the dispositionist lay theory tapped by the SESQ is evident even on implicit measures.

One might expect that SESQ historicism scores should predict SSIs, but we were not so sure. The reason has to do with the specific content of the situational explanations used by Ham and Vonk (2003), whose stimuli we use here. Specifically, the SSI stimuli from Ham and Vonk (2003) refer primarily to properties of objects with which an actor has a momentary interaction (e.g., *the fence was low*; *the boulder was light*). In contrast, historicism on the SESQ concerns “nurture” beliefs (e.g., *parents and community have shaped the type of person she is*). It seems to us that these two notions of “external causes” are so different that historicism scores might well be unrelated to SSIs.

### Method

#### Participants

One-hundred fifteen participants (59 female) participated for course credit.

#### Procedure

After signing a written consent form, participants completed the SESQ at a pretest. A few weeks later, they returned individually to take part in an ostensibly unrelated study. Participants were randomly assigned to either the *STI condition* or *SSI condition* and were seated at a computer terminal. After signing a written consent form, participants received instructions indicating that their task would consist of reading behavior descriptions displayed on the screen and then indicating as quickly and accurately as possible whether or not a word displayed immediately afterward had actually appeared in the behavior description. To motivate participants, a reward of $20 was promised to the participant whose responses were the fastest and most accurate. Participants were instructed to press the “a” key if the probe word had actually appeared in the behavior description and to press the “6” key on the number pad if the probe word had not actually appeared in the behavior description. They were also instructed to keep their left and right index fingers on the keys during the entire task. Participants completed 12 practice trials to familiarize them with the task.

Next, they began the actual task. On each trial, a row of X's was presented in the center of the screen for 1000 ms. Then, a behavior description was displayed for 3000 ms followed by a blank screen which lasted for 500 ms. A row of X's then appeared for 500 ms, followed by a probe word. The probe word remained on the screen until participants indicated “Yes” or “No” (*Was this word actually in the sentence you just read*?). Once an answer was provided, a blank screen was displayed for 1000 ms. Then the next trial began. There were 10 critical trials on which the probe word was implied by the preceding behavior description. There were also 10 control trials on which the probe word was not implied by the preceding behavior description. The critical and control trials consisted of the same behavior descriptions and probe words, but in the control trials the probes were rearranged to be irrelevant to the behavior descriptions. Although the 10 behavior descriptions were always the same, in the STI condition the probe words were traits whereas in the SSI condition probe words were features of the situation. Forty filler trials were also included and these served to add trials on which “yes” was the correct response and on which participants needed to attend to the verb in the behavior description; see Ham & Vonk [69] for rationale. The 60 combinations of behavior descriptions and probes were presented in random order. Response times (RT) and correctness were recorded by the computer.

### Results and Discussion

#### Reaction times

Overall, error rates were very low (*M* = 1.01%, *SD*  = .02, Range  = 0 to 5%). RTs were analyzed only if the response had been correct. Because analyses of RTs can be very sensitive to outliers, we analyzed our response time data using two methods. First, we employed an absolute cutoff criterion of <200 ms and >2000 ms. Using this cutoff criterion resulted in the deletion of only .28% of responses. Second, because the RT data were positively skewed, we performed a natural log transformation. Results using either approach were virtually identical. For ease of interpretation, the analyses presented below are based on the cutoff criterion.

Prior to testing our primary hypotheses, we computed a 2(Trial Type: control, critical) X 2(Probe Type: trait, situation) between-within ANOVA. This revealed a main effect of Trial Type, *F*(113)  = 9.77, *p* = .002, which reflected that fact that RTs were slower on critical trials (*M* = 657) than on control trials (*M* = 637). Neither the main effect of Probe Type nor the interaction was significant, *F*s<1.83. This replicates the findings of Ham and Vonk and suggests that participants were making both STIs and SSIs in our study.

Next, to test our hypotheses regarding dispositionism, we followed the recommendations of Judd, Kenny, and McClelland [72] for testing moderation in a within-subjects design. First, we transformed the within-subjects variable of trial type (critical vs. control) into a difference score that represented the difference between RTs on critical versus control trials. This was done such that higher scores indicated slower RT on critical as compared to control, suggesting the presence of a spontaneous inference. Thus, we will hereafter call this variable *spontaneous inferences*.

**Figure 1:**
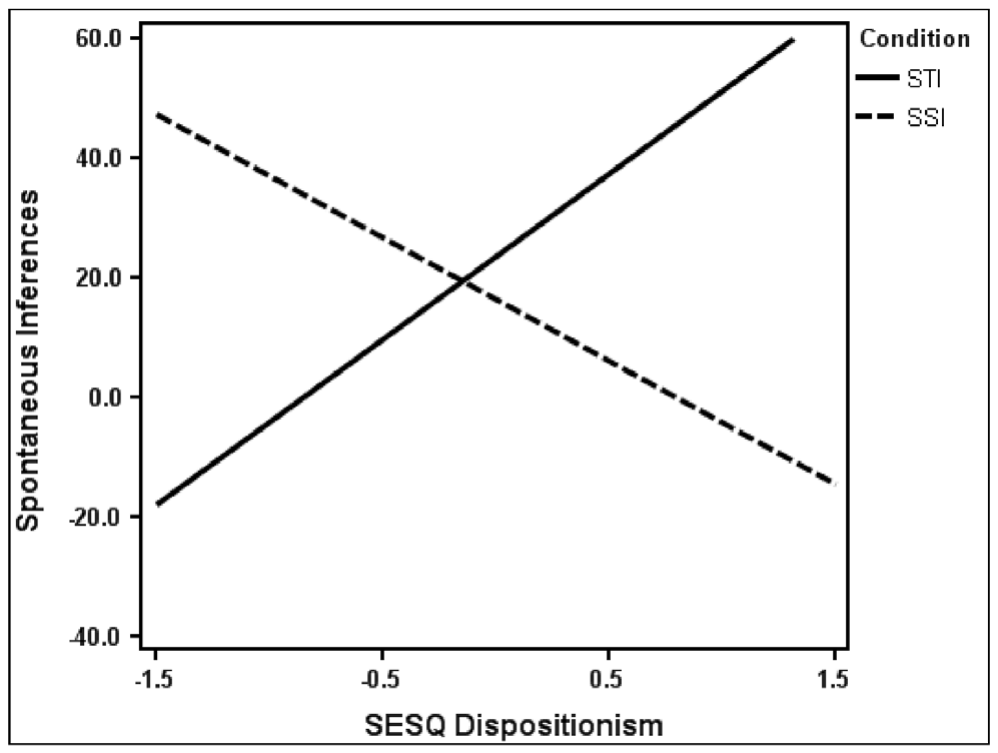
SESQ dispositionism and spontaneous inferences. SESQ dispositionism strongly predicts increased levels of spontaneous trait inference (STI) and weakly predicts decreased levels of spontaneous situation inference (SSI).

We regressed the spontaneous inference variable on condition (STI vs. SSI), SESQ dispositionism, SESQ historicism, and all their 2- and 3-way interactions (the version of the SESQ used in this study omitted the controllability dimension). This analysis revealed only the predicted condition by SESQ dispositionism interaction, *t*(107) = 2.93, *p* = .004 (all other *t*s <|1.19|, *p*s>.24). Follow-up simple slope analyses—plotting the relation between dispositionism and spontaneous inferences separately for the STI and SSI conditions—were computed. See Figure 1. In the STI condition, we found a significant positive relation between SESQ dispositionism and spontaneous inferences, *t*(111) = 2.33, *p* = .02 (*d* = .44). In the SSI condition, there was a slight reversal of this pattern which did not attain statistical significance, *t*(111) = −1.47, *p* = .15 (*d* = .28). Thus, SESQ dispositionism scores predicted an increased tendency to make spontaneous trait inferences, and were weakly predictive of a reduced tendency to make spontaneous situation inferences. As noted earlier, we questioned whether SESQ historicism would predict SSIs, and the evidence suggests that they did not. Validity evidence for SESQ historicism comes next.

## Study 5: SESQ Historicism and the Fundamental Attribution Error

In Study 5, we examine the predictive validity of the historicism dimension of the SESQ. To do so, we examine whether historicism moderates one of the most “fundamental” phenomena of social cognition: The *Fundamental Attribution Error* (*FAE*), which is the tendency to neglect or underweight external causes when making social judgments [24], [27], [73]. To test this, we performed a conceptual replication of Jones and Harris's [24] classic study.

We predicted that SESQ dispositionism would not moderate the tendency to commit the FAE. Given that lay dispositionism is a frequently given explanation for the FAE [27], our prediction deserves some comment. A careful reading of Gilbert and Malone's analysis [27] (see especially pp. 22–24) shows that it is not belief in dispositions *per se* that fosters the FAE. Rather, it is either blindness about or explicit dismissal of the “power of the situation” in shaping actions and dispositions. As noted above, dispositionism is independent of belief in the power of the situation [27] (see also Study 1 regarding the lack of correlation between dispositionism and historicism). Thus, we expect that dispositionism will not moderate the FAE, but rather it will be those who *explicitly affirm the power of external social forces to mold the individual* (i.e., high historicism) who will be less prone to committing the FAE.

### Method

#### Participants

Eighty-nine participants (43 female) participated for credit in their introductory psychology course.

#### Procedure

After signing a written consent form, participants completed the SESQ at a pretest. A few weeks later, they returned to a different location in small groups, met a different experimenter, signed a written consent form, and completed an experiment modeled after Jones and Harris. Specifically, they learned that they would be reading an essay about Affirmative Action (AA) written by a college student, Pat O'Malley, for a journalism class. Participants were randomly assigned to either the *no choice condition* or the *choice condition* and to the *anti-AA condition* or the *pro-AA condition*.

Participants in the no choice condition learned that “the professor told each student which position to defend. Thus, Pat did not choose which position to argue.” Participants in the choice condition learned that “the professor allowed each student to choose which position to defend. Thus, Pat chose to argue the position in the essay you'll read.” Next, participants in the anti-AA condition read an essay ostensibly written by Pat that provided several arguments against Affirmative Action, whereas participants in the pro-AA condition read an essay ostensibly written by Pat that provided several arguments in favor of Affirmative Action. Finally, participants were asked to diagnose Pat's “true, personal attitude” on two 9-point scales with endpoints labeled *Definitely Opposes Affirmative Action* and *Definitely Supports Affirmative Action* or *Definitely Against Affirmative Action* and *Definitely In Favor of Affirmative Action*. These two items were highly correlated (*r* = .92), and thus were averaged to form an index of the extent to which Pat was perceived as favorable toward AA (*M* = 4.57, *SD*  = 2.39).

### Results and Discussion

Ratings of Pat's attitude were regressed on the choice manipulation, the position manipulation (anti or pro), SESQ historicism, SESQ dispositionism, and all their 2-, 3-, and 4-way interactions (again, the controllability dimension was omitted on the version of the SESQ used in this study). The analysis revealed a main effect of position, *t*(73)  = 8.83, *p*<.001 (*d* = 2.1), with those in the pro-AA condition perceiving Pat as more favorable to Affirmative Action than those in the anti-AA condition. There was a marginal and not particularly intelligible main effect of dispositionism, *t*(73)  = 1.67, *p* = .10, suggesting that those who scored high in dispositionism tended to rate Pat as more favorable toward Affirmative Action. More importantly, replicating Jones and Harris, there was a position by choice interaction, *t*(73) = 4.00, *p*<.001. The nature of this was such that the effect of position on ratings of Pat's attitude was stronger in the choice condition, *t*(84) = 9.29, *p*<.001 (*d* = 2.0), than in the no choice condition, *t*(84) = 3.83, *p*<.001 (*d* = .84). Notably, the fact that there remained a significant difference between the anti- and pro-AA conditions *even in the no choice condition* replicates the evidence for the FAE found by Jones and Harris.

**Figure 2:**
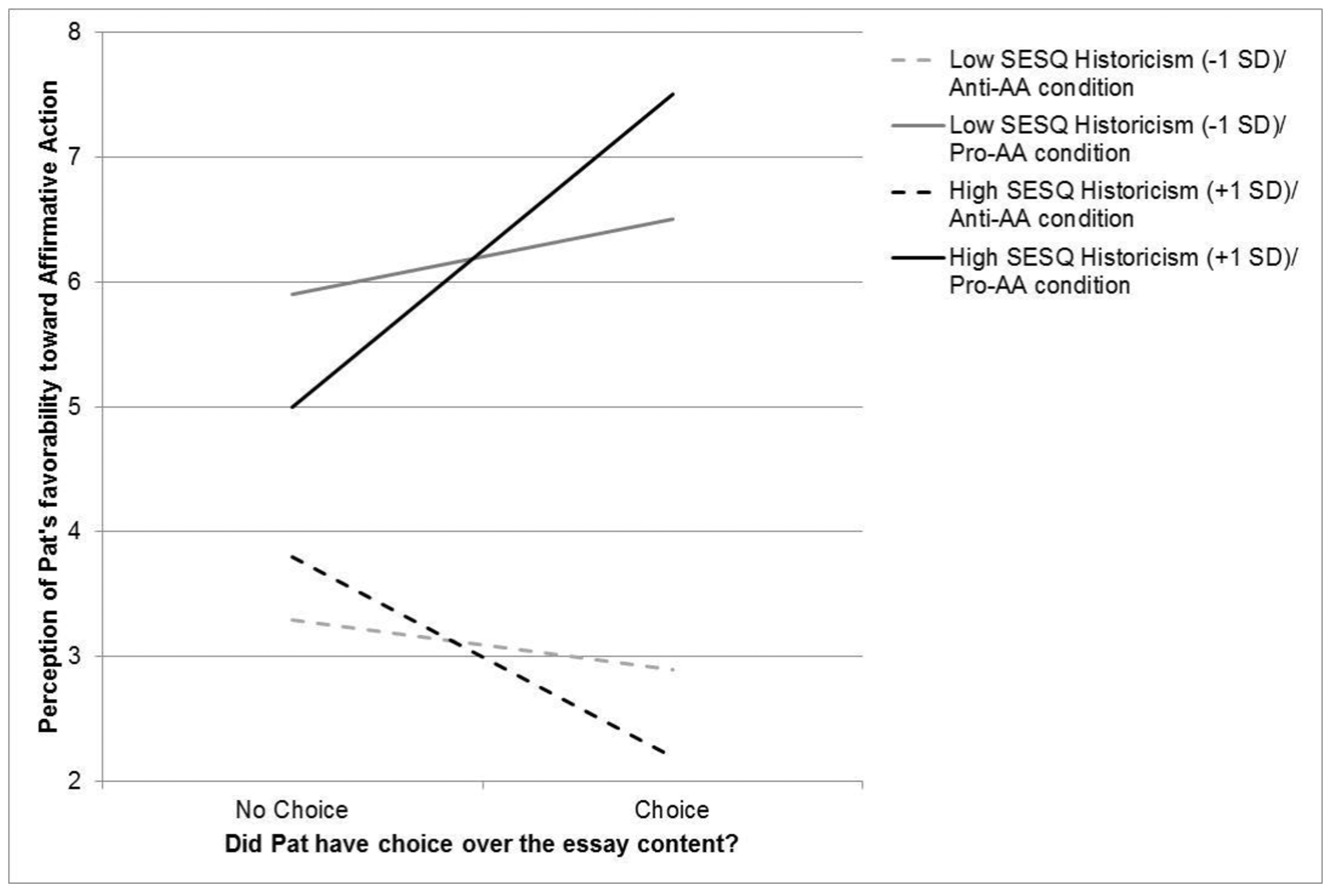
SESQ historicism and the Fundamental Attribution Error. SESQ historicism predicts increased sensitivity to the choice manipulation of the classic Jones and Harris (1967) paradigm, and thus a reduced tendency to commit the FAE.

Going beyond Jones and Harris, however, our analysis also revealed that this two-way interaction was moderated by SESQ historicism, creating a three-way interaction, *t*(73) = 2.33, *p* = .023. As can be seen in Figure 2, this interaction was driven by the fact that, as predicted, those high in SESQ historicism were more strongly affected by the choice manipulation than were those low in historicism. Indeed, in the anti-AA condition, those high (+1 SD) in historicism rated Pat as more opposed to Affirmative Action when Pat had choice than when Pat had no choice, *t*(41) = −2.27, *p* = .028 (*d* = .71), whereas the choice manipulation made no difference to those low (−1 SD) in historicism, *t*<1. Similarly, in the pro-AA condition, those high (+1 SD) in historicism rated Pat as more in favor of Affirmative Action when Pat had choice than when Pat had no choice, *t*(39) = 3.31, p = .002 (*d* = 1.06), whereas the choice manipulation made no difference to those low (−1 SD) in historicism, *t* = 1.12, *p* = .27.

Another way of parsing these data is to look specifically at judgments within the no choice condition, in which, logically, the pro- and anti-AA essay writers should receive similar ratings because of the professor's strong influence on their attitude expressions. Examining that condition reveals that those high in historicism came quite close to avoiding the FAE altogether. Specifically, in the no choice condition those high in historicism (+1 SD) showed only a marginal tendency to rate Pat's attitude differently in the anti-AA (*M* = 3.8) as compared to the pro-AA condition (*M* = 5.0), *t*(39) = 1.77, *p* = .08 (*d* = .57). In contrast, this same difference was twice as large among those low (−1 SD) in historicism (*M*s = 3.3 and 5.9, respectively; *t*(39) = 3.57, *p* = .001; *d* = 1.14).

## Study 6: SESQ Controllability and the Polarization of Moral Judgments

In Study 6, we focus on the controllability dimension of the SESQ. The study is based on Weiner's [8] insight that controllability perceptions play a crucial role in moral blame. In particular, Weiner et al. [7] showed that when a stigmatizing condition is seen as controllable by its possessor, blame is amplified. In contrast, when a stigmatizing condition is seen as uncontrollable by its possessor, blame is inhibited.

Study 6 will examine whether those with a strong general belief in controllability (according to the SESQ) tend to perceive relatively controllable stigmas as *highly* controllable, and thus to show heightened blame compared to those with a weak general belief in controllability. We will also include a condition in which participants respond to relatively uncontrollable stigmas. We are interested in the possibility of a contrast effect: When a stigma is uncontrollable, will those with strong general belief in controllability perceive that stigma as *highly un*controllable because the stigma contrasts so markedly with their generally high control expectations? Such a contrast effect would ironically produce attenuated blame responses among those with strong general belief in controllability. In effect, then, Study 6 explores whether the controllability dimension of social explanatory style predicts polarization—both amplification and attenuation—of blame responses.

### Method

#### Participants

Sixty-seven participants (44 female) participated for credit in their introductory psychology course.

#### Procedure

After signing a written consent form, participants completed the SESQ at a pretest. A few weeks later, they returned to a different location and met a different experimenter. At this follow-up session, after signing a written consent form, they rated four stigmatized categories taken from Weiner et al. [7]. Based on pilot testing, we chose the two categories that were rated highest in controllability (*controllable stigmas*: *Child Abusers, The Obese*) and the two categories that were rated lowest in controllability (*uncontrollable stigmas*: *People with Alzheimer*'*s, The Blind*). Participants in the present study rated the two controllable and the two uncontrollable stigmas in terms of controllability (e.g., *Did a child abuser have control over whether he/she became a child abuser*?). Responses were made on a 9-point scale with endpoints labeled *No Control At All* (1) and *Total Control* (9). Participants also rated how much blame they assigned to each stigma group (e.g., *To what extent do you blame a child abuser*?). Responses were made on a 9-point scale with endpoints labeled *No Blame* (1) and *Total Blame* (9). The order in which these ratings were made was counter-balanced across participants. There were no order effects. To prepare these ratings for analysis, we averaged the controllability ratings for the two controllable stigmas (*M* = 7.45, *SD* = 1.24) and for the two uncontrollable stigmas (*M* = 1.75, *SD* = .84). Next, we averaged the blame ratings for the two controllable stigmas (*M* = 7.03, *SD* = 1.22) and for the two uncontrollable stigmas (*M* = 1.28, *SD* = .47).

### Results and Discussion

Our primary hypotheses were that SESQ controllability scores would predict heightened perceptions of control over controllable stigmas (an assimilation effect) and perhaps also attenuated perceptions of control over uncontrollable stigmas (a contrast effect). These alterations in perceived control should, in turn, translate into heightened blame for controllable stigmas and attenuated blame for uncontrollable stigmas.

To test these possibilities, we first looked at perceived control ratings. Specifically, we computed a repeated-measures ANCOVA (repeated measure: controllability ratings for controllable stigmas, controllability ratings for uncontrollable stigmas) with SESQ controllability as a continuous covariate. This analysis revealed a significant interaction, *F*(1, 65) = 23.54, *p*<.001. We calculated predicted means for high and low levels of SESQ controllability and the pattern of means fit our predictions. Specifically, perceived control over controllable stigmas was higher among those with strong general belief in controllability (*M*_controllable_ = 8.0 at +1 SD of SESQ controllability) than among those with weak general belief in controllability (*M*_controllable_  = 6.84 at −1 SD of SESQ controllability). And, in contrast, perceived control over uncontrollable stigmas was *lower* among those with strong general belief in controllability (*M*_uncontrollable_  = 1.50 at −1 SD of SESQ controllability) than among those with weak general belief in controllability (*M*_uncontrollable_  = 1.99).

Next, we examined blame ratings in the same repeated-measures ANCOVA. Once again, the interaction with SESQ controllability was significant, *F*(1, 65) = 32.47, *p*<.001. Once again, we calculated predicted means at high and low levels of SESQ controllability and the pattern of means fit our predictions. Specifically, blame for controllable stigmas was higher among those with strong general belief in controllability (*M*_controllable_  = 7.58 at +1 SD of SESQ controllability) than among those with weak general belief in controllability (*M*_controllable_  = 6.39 at −1 SD of SESQ controllability). And, in contrast, blame for uncontrollable stigmas was *lower* among those with strong general belief in controllability (*M*_uncontrollable_  = 1.04 at −1 SD of SESQ controllability) than among those with weak general belief in controllability (*M*_uncontrollable_  = 1.53 at −1 SD of SESQ controllability).

**Figure 3:**
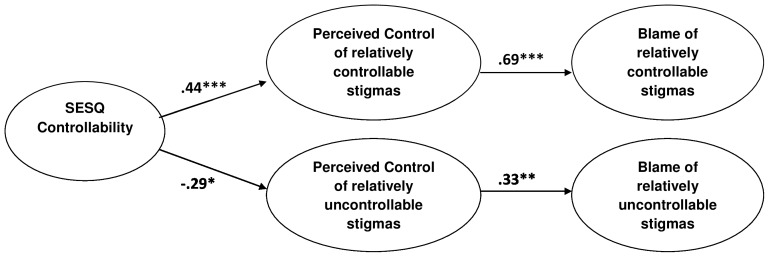
SESQ controllability and blame. SESQ controllability predicts polarization of moral judgments. Among those scoring high on SESQ controllability, perceived control and blame are intensified for relatively controllable stigmas, but attenuated for relatively uncontrollable stigmas. *p<.05, **p<.01, ***p<.001.

To capture this blame polarization process in a single analysis, we computed the path model depicted in Figure 3. The model assumes that general belief in controllability (from the SESQ) affects perceived control over both controllable and uncontrollable stigmas, and these controllability perceptions, in turn, contribute to blame. Because they are correlational, of course, the present data cannot rule in any pattern of causal relations. We note, however, that prior literature provides overwhelming evidence for a causal effect of lay theories on perceptions of specific instances [51], [74], [75], [76], [77] and also for a causal effect of perceived control on blame [8]. As can be seen in Figure 3, for controllable stigmas, SESQ controllability scores significantly predicted heightened perceptions of control and these heightened perceptions of control, in turn, significantly predicted heightened blame. In contrast, for uncontrollable stigmas, SESQ controllability scores significantly predicted attenuated perceptions of control and these attenuated perceptions of control, in turn, significantly predicted attenuated blame.

Elsewhere [4] we suggest that historicism can foster compassionate, non-blaming responses. Thus, we examined the relation of SESQ historicism to blaming of the stigma categories. In regression analyses that also included SESQ controllability scores as a predictor, we found that SESQ historicism predicted less blaming of controllable stigmas, t(64) = −2.90, p = .005 (β = −.31). In contrast, SESQ historicism was unrelated to blaming of uncontrollable stigmas, *t*<1.

The key finding here is that general belief in controllability—as tapped by the SESQ—is associated with polarized moral blame: Intensifying perceived control and blame for relatively controllable stigmas, and attenuating perceived control and blame for relatively uncontrollable stigmas. Also worthy of note is that SESQ historicism—independent of SESQ controllability—predicts less blame of controllable stigmas.

## General Discussion

Explanation pervades social life. Furthermore, the explanations people generate powerfully shape their social emotions and social interactions. How do people select an explanation for an observed social event? Early work suggested that they rely on a logical reasoning process [29], [30], and subsequent work suggested that they do whatever is cognitively easiest [33]. More recent work focuses on the perceiver's pre-existing general theories [40], [43], [44]. Our work grows out of this latter tradition. We suggest that—based on cultural, educational, and personal experiences, filtered through one's cognitive and motivational style—individuals develop various general beliefs regarding the causes of social events. We refer to these beliefs as social explanatory styles. Our main goal in the present article was to offer a measure of individual differences in three dimensions of social explanatory styles, along with separate validity evidence for each of these dimensions.

Accordingly, we have offered the Social Explanatory Styles Questionnaire (SESQ). Rooted in classic theorizing, the SESQ measures three independent dimensions of social explanatory styles: Dispositionism, historicism, and controllability. Studies 1–3 examined basic psychometric properties of the SESQ along with its relations to other relevant constructs (to establish divergent validity). Study 1 revealed that each dimension of the SESQ shows strong internal consistency. Also, based on both factor analysis and correlational analyses, Study 1 suggested that the three SESQ dimensions are largely unrelated to each other. Study 2 provided evidence that the SESQ dimensions are independent of political ideology (RWA, SDO) and only modestly related (in sensible ways) to the Big Five traits and to emotion regulation. Study 2 also suggested that those high in Need for Cognitive Closure are more dispositional and less historicist in their social explanatory styles. Finally, Study 3 looked at relations between the SESQ and other lay theories constructs: Implicit person theories, implicit trait beliefs, and implicit context beliefs. Results indicated that SESQ historicism and SESQ controllability are essentially unrelated to any of these other lay theories measures. Thus, the majority of information provided by the SESQ is not provided by other measures. In contrast, SESQ dispositionism is moderately (not highly) related to the implicit trait beliefs construct offered by Church, Ortiz, and their colleagues [50]. Future work is needed to more precisely understand the differences in what is being assessed by the SESQ dispositionism subscale and the implicit trait beliefs measure.

Do the three dimensions of the SESQ have predictive validity in relation to important social cognitive phenomena? Yes, they do. This was the focus of Studies 4–6. In Study 4, we examined whether SESQ dispositionism moderates the tendency to make spontaneous trait inferences (STIs). Given that STIs can affect downstream consequences such as impression formation and approach/avoidance tendencies, it is important to understand when they will be made. Study 4 presented evidence that STIs are especially likely to be made by perceivers with a strong belief in dispositionism. In Study 5, we examined whether SESQ historicism moderates the tendency to commit the Fundamental Attribution Error. We found that it did, with high scorers on the SESQ historicism dimension being significantly less likely to commit the FAE than were low scorers. Finally, in Study 6, we examined whether general controllability beliefs would moderate blame judgments. Study 6 revealed that, interestingly, SESQ controllability predicted polarization of blame judgments: Heightened perceptions of control and blameworthiness for controllable stigmas, and attenuated perceptions of control and blameworthiness for uncontrollable stigmas. Thus, general controllability beliefs might paradoxically contribute to both condemnation and compassion toward others, a possibility surely worthy of further exploration. Indeed, existing work tends to emphasize the negative, condemnatory side of controllability beliefs [7], [57]. SESQ historicism also predicted reduced blame of those with controllable stigmas, independent of SESQ controllability scores.

In sum, Studies 4–6 provide predictive validity evidence regarding the three fundamental dimensions of social explanatory styles tapped by the SESQ. It is worth noting that SESQ scores in Studies 4–6 were always obtained several weeks prior to the main dependent variables, suggesting that social explanatory styles are robust and stable dimensions of people's thinking.

Where do we go from here? First, as noted above, we expect that social explanatory styles develop from a variety of influences including culture, socialization experiences, extrapolation from the self, general cognitive styles, and so on. Future work is needed to examine the contributions of these various factors to the development and modification of the three social explanatory dimensions tapped by the SESQ. Such work will be especially valuable if social explanatory styles are eventually shown to have the types of pervasive effects on social responding that we expect they will have.

Furthermore, we anticipate that, as with the Attributional Style Questionnaire [21], future research will suggest that certain configurations of explanatory beliefs—interactive combinations of the three basic explanatory dimensions—are particularly important. Researchers should therefore think in terms of interactions among the SESQ dimensions when attempting to understand social phenomena. Indeed, the fact that we independently validated each subscale of the SESQ should not be taken to mean that those subscales should always be treated separately in future theorizing and research. In fact, unpublished data from our lab already highlight the importance of interactions among social explanatory style dimensions for predicting implicit blame. In one study, we had participants learn about a violent criminal. Immediately afterwards, we assessed the extent to which blame-relevant constructs were automatically activated by priming with the criminal's name. The theoretical question was whether implicit blame is impacted by social explanatory styles in the same manner as explicit blame (as in Study 6 above), or whether, alternatively, implicit blame shows a different pattern than explicit blame [e.g., everyone experiences blame at the implicit level, but certain people—based on their general beliefs in controllability and historicism (see Study 6)—adjust their explicit judgments towards reduced blame]. We found an interaction between historicism and controllability beliefs (the same dimensions relevant for predicting explicit blame; see Study 6) for predicting implicit blame: Those who score both high on historicism *and* low on controllability show especially low levels of implicit blame. In addition to showing interactive effects of social explanatory style dimensions, this study also highlights how social explanatory styles shape immediate, uncontrollable responses to social stimuli (see also Study 4 above).

What other types of effects on social responding should be explored? We have an enduring interest in moral emotions, especially prosocial moral emotions that reflect concern for others (e.g., compunction regarding mistreatment of others and compassion for the plight of others; [4] [11], [18], [78]). Thus, an ongoing program of research in our labs involves connecting social explanatory styles to enduring moral emotional tendencies. To date, we have shown that the SESQ historicism predicts dispositional compassion-proneness and that historicism has a causal impact on compassionate responding [18]. One issue that remains to be investigated is why historicism has these effects. Gill et al. [4] offer a model in which historicist explanations for negative aspects of a target increase the sense that the target has suffered, and perceived suffering mediates the impact of historicist explanations on compassion. Gill et al. examined their model in the context of intergroup attitudes and not general explanatory styles. Thus, a task for future research is to examine whether a general belief that suffering lies behind people's negative acts and outcomes mediates the connection between SESQ historicism and dispositional compassion-proneness. More broadly, research on social explanatory styles needs to move beyond predicting personality self-reports (e.g., compassion-proneness) and responses to verbal stimuli in the lab (as in Studies 4–6 above). Researchers should examine whether social explanatory styles predict patterns of responding in face-to-face interactions with novel social partners, emotional responding within enduring relationships, patterns of blame vs. compassion reflected in social policy positions, and so on. We are examining all these domains of social life in our labs. Also, more work is needed regarding causal effects of social explanatory styles, although, of course, it is no easy task to manipulate *general* beliefs in social causality.

We will conclude by noting the massive body of work supporting the importance of self-oriented explanatory style in shaping self-relevant cognitive, emotional, and behavioral outcomes [20]. Explanatory style—an interactive combination internality, globality, and stability beliefs—is most famously linked to the development of depression [22], [79]. Beyond this, however, it has also been linked to performance in law school [80], productivity in the workplace [81], athletic performance [82], tendency toward risky aggression [83], and even physical health [84]. If explanatory style regarding the self is such a potent predictor of diverse areas of self-functioning, then it is plausible that social explanatory styles—perhaps including particular, interactive configurations of basic social explanatory dimensions—will be a similarly potent predictor of diverse areas of social functioning. We believe that this is a possibility worthy of exploration and we hope the SESQ will be a useful tool in that endeavor.

## Supporting Information

Appendix S1 **The Social Explanatory Styles Questionnaire (SESQ).**
(DOCX)
