# Supplementary material for: The Social Explanatory Styles Questionnaire: Assessing Moderators of Basic Social-Cognitive Phenomena Including Spontaneous Trait Inference, the Fundamental Attribution Error, and Moral Blame
Source: PLoS One. 2014 Jul 9;9(7):e100886. doi: 10.1371/journal.pone.0100886 (PMC4090131; doi:10.1371/journal.pone.0100886)
Supplement: Appendix S1 — The Social Explanatory Styles Questionnaire (SESQ). (DOCX) [file pone.0100886.s001.docx]

**Appendix S1: The Social Explanatory Styles Questionnaire (SESQ)**

**Sarah often ridicules and belittles her children. She tells them they are lazy, sloppy, and even "worthless".**

**WHY has Sarah become such a cruel mother?**

(1) A major factor is Sarah’s **character traits**

. **1-----------2-----------3-----------4-----------5**

**NO YES**

(2) A major factor is Sarah’s **external circumstances/life experiences**.

**1-----------2-----------3-----------4-----------5**

**NO YES**

(3) Sarah has **control** over her cruelty.

**1-----------2-----------3-----------4-----------5**

**NO YES**

Other SESQ items (in order):

- Steven never tries to take another’s perspective. When he disagrees with someone, he is stubborn, angry, and insulting.
- Janet, a successful accountant, volunteers at a local Food Bank for 6-10 hours per week.
- Robert has been arrested numerous times for "petty offenses" since he was 14.
- Susan exudes love for others. People look forward to being with her because she shares so much kindness and inspiration.
- Bill is very generous with his time and knowledge, patiently helping others even when it is inconvenient for him.
- James worked tirelessly to start a number of local Community Centers that provide a variety of activities for community members to enjoy.
- Beth sometimes has sexual relations with other men while her husband is out of town.
